# Engineering Magnetic Anisotropy of Rhenium Atom in Nitrogenized Divacancy of Graphene

**DOI:** 10.3390/nano13050829

**Published:** 2023-02-23

**Authors:** Honglei Liu, Guangtian Ji, Pingji Ge, Guixian Ge, Xiaodong Yang, Jinli Zhang

**Affiliations:** 1 Key Laboratory for Green Processing of Chemical Engineering of Xinjiang Bingtuan, School of Chemistry and Chemical Engineering, Shihezi University, Shihezi 832003, China; 2 Xinjiang Production & Construction Corps Key Laboratory of Advanced Energy Storage Materials and Technology and Department of Physics, College of Science, Shihezi University, Shihezi 832003, China; 3 School of Chemical Engineering and Technology, Tianjin University, Tianjin 300072, China

**Keywords:** density functional calculations, magnetic anisotropy, electronic properties

## Abstract

The effects of charging on the magnetic anisotropy energy (MAE) of rhenium atom in nitrogenized-divacancy graphene (Re@NDV) are investigated using density functional theory (DFT) calculations. High-stability and large MAE of 71.2 meV are found in Re@NDV. The more exciting finding is that the magnitude of MAE of a system can be tuned by charge injection. Moreover, the easy magnetization direction of a system may also be controlled by charge injection. The controllable MAE of a system is attributed to the critical variation in d_z_^2^ and d_yz_ of Re under charge injection. Our results show that Re@NDV is very promising in high-performance magnetic storage and spintronics devices.

## 1. Introduction

A fundamental obstacle to the downscaling of spintronics devices is the spin-reorientation transition (SRT), induced by thermal fluctuations at room temperature. To prevent the occurrence of SRT, magnetic materials should possess large magnetic anisotropy energy (MAE) [1,2,3]. Therefore, the search for magnetic materials possessing large MAE is of both fundamental interest and technological merit for future spintronics device applications. In general, MAE originates in large spin–orbit coupling (SOC) interactions. Recently, the two-dimensional materials containing 5d transition-metal (TM) atoms have been paid more attention in magnetic storage areas, due to strong SOC in 5d TM atoms [4,5,6,7,8,9,10,11,12].

To stabilize a magnetic bit against thermal agitation, a material is required to possess large MAE. Meanwhile, to reduce energy consumption, a material is desired to have low MAE in the process of writing information. To solve this confliction, the MAE of magnetic materials should be manipulated. Previous studies have suggested that the MAE of magnetic materials can be controlled by an electric field [13,14,15,16,17], surface charging [11,18,19,20], and biaxial strain [8,15,21,22,23,24,25]. Experimental studies demonstrated that the electric field can manipulate magnetization orientation of a ferromagnetic semiconductor [26]. First principles calculations predicted that MAE could be tuned in an Fe(001) monolayer by an applied electric field [27]. A recent study of Fe-Pt multilayers suggested that the magnitude of MAE and the magnetization direction can be controlled simultaneously by surface charging [18].

Although there are extensive investigations on the modulation of MAE of magnetic materials, few stable materials that have large MAE and are easily affected by ambient conditions have been found so far. Recent studies have indicated that graphene-based materials can be easily tuned by external conditions [7,28,29]. To firm the TM atom and reduce the probability of forming cluster [30,31], one may introduce defects, such as single vacancies and nitrogenized divacancies, into graphene [12,32,33,34]. In this work, we investigate the stability, electronic and magnetic properties of rhenium atom in nitrogenized divacancy of graphene (Re@NDV). We found that Re@NDV is highly stable and possesses large MAE of 71.2 meV. Moreover, the MAE of Re@NDV can be manipulated by charge injection. The physical mechanism of tunable MAE is discussed. The charge injection not only changes the intensity of Re@NDV MAE, but it also changes the direction of the easily magnetized axis. The control of the easily magnetized axis is beneficial to information storage and erasure. We hope that our computational results can help future experimental scientists synthesize two-dimensional Re@NDV materials. Additionally, we also consider some new materials as candidates for spintronic devices [35,36,37,38,39].

## 2. Computational Methods

The structural and electric properties were calculated using the density functional theory (DFT) in the Vienna ab initio simulation package (VASP) [40,41], the VASP is a simulation software package developed by Hafner’s group at the University of Vienna. We used VASP version 5.5.4. The generalized gradient approximation of Perdew–Burke–Ernzerhof (PBE) was used [42]. The interactions between electrons and nuclei were described by the projector augmented wave (PAW) method [43]. The kinetic energy cutoff of the plane wave is 500 eV. A 3 × 3 × 1 k-point mesh was adopted to relax the supercell. Geometric structural relaxations were performed until residual force per atom was smaller than 0.01 eV/Å. A 6 × 6 supercell was chosen along the x and y directions of graphene layer. The vacuum region of 15 Å perpendicular to the NDV of Re@NDV was built.

The MAE value was calculated by a noncollinear mode [44]. The atomic positions were fully relaxed until the total energy was converged to smaller than 10^−7^ eV. MAE is defined as $\mathrm{MAE}=E_{z}-E_{x/y}$, where $E_{z}$ and $E_{x/y}$ are the total energies for the *z* axis and *x*/*y* axis magnetizations, respectively. The positive and negative MAEs indicate the easy axis, parallel and perpendicular to the graphene plane, respectively.

## 3. Results and Discussions

### 3.1. Structure and Stability of Re@NDV

**Figure 1:**
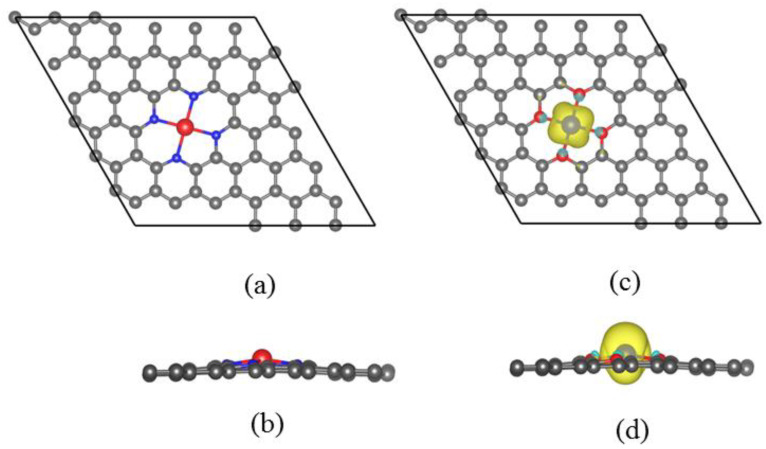
(**a**,**b**) Structure diagrams of Re@NDV; (**c**,**d**) spin density diagrams of Re@NDV. In (**a**,**b**), blue and red represent N atoms and Re atoms, respectively. In (**c**,**d**), yellow represents spin-up charge density, cyan represents spin-down charge density, and red represents N atoms. (**a**,**c**) Top view; (**b**,**d**) side view.

We first investigated the optimized structure of Re@NDV, as displayed in Figure 1a,b. The optimized stable structure of Re@NDV involved the Re atom located on the top site of NDV. The distance between Re and NDV was 0.303 Å, which is much lower than that of Re@Gr (1.686 Å). To examine the stability of the Re atom at the nitrogenized divacancy, we estimated the binding energy, which is expressed as:

(1) Eb=−ERe@NDV+ERe+ENDV

where E[Re@NDV], E[Re] and E[NDV] represent the total energies of Re@NDV, the Re atom and NDV, respectively. The large binding energy of 6.94 eV (see Table 1) and the short distance between Re and N atoms (0.30 Å) indicate that the Re atom can be strongly stable on the NDV surface.

**Figure 2:**
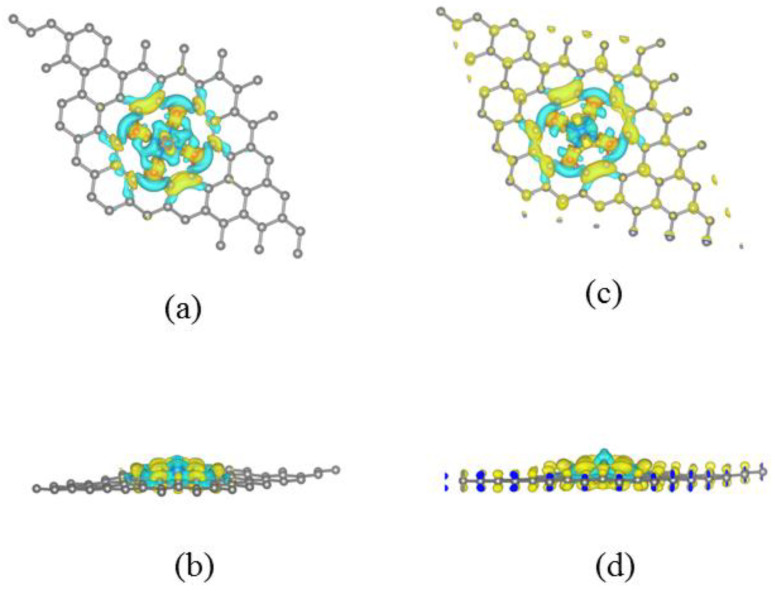
Charge density difference isosurfaces of Re@NDV under neutral and adding 0.2e/unit cell, respectively. (**a**,**c**) Top view; (**b**,**d**) side view. The yellow color represents charge accumulation, while the cyan color represents the charge depletion zone. ∆ρ=ρRe@NDV−Gr−ρRe−ρNDV−Gr.

To illuminate the strong interaction between Re and NDV, Figure 2a,b present the charge density difference for Re@NDV. One can see that electrons deplete from the Re atom and accumulate in regions between the Re–N bonds. This indicates that there is a covalent interaction between Re and N atoms, resulting in strong binding between Re and NDV. The Bader charge of Re atom amounted to 1.36e (see Table 1), suggesting that the electron transfers from Re to N atoms, which is consistent with the results of charge density difference.

.

### 3.2. Magnetic Properties of Re@NDV

We now discuss the magnetic properties of Re@NDV. As shown in Table 1, Re@NDV has a large spin magnetic moment of 3.02μ_B_. Figure 1c,d display the spin density of Re@NDV. It can be seen that the spin density of Re@NDV mostly comes from the Re atom. This suggests that the Re atom provides a major magnetic moment of Re@NDV. Furthermore, adsorption of the Re atom to NDV induces strong magnetocrystalline anisotropy with MAE values of 71.20 meV and an easy axis along the *y* axis. This value is far greater than that of Re atom adsorption on divacancy-defect graphene (DV) (about 2.67 meV), which is derived from the increase in the magnetic moment of the Re atom in Re@NDV. Re@NDV structure with high stability and large MAE has potential applications in information storage.

### 3.3. Charge Manipulation of MAE in Re@NDV

**Figure 3:**
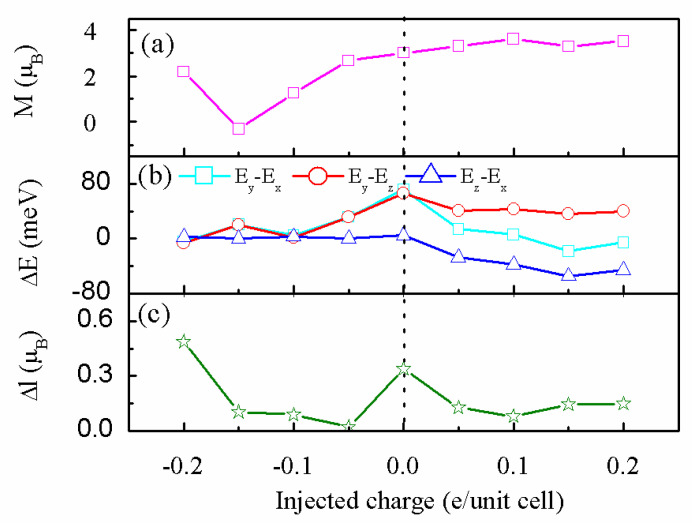
Injected charge dependence of (**a**) magnetic moment and (**b**) energy difference between each magnetization direction. (**c**)The difference in orbital magnetic moment between easy and hard magnetization directions for Re of Re@NDV. The charge-doping scale (in units of e/unit cell) is referred to as the neutral system. Positive (negative) values stand for an excess (lack) of valence electrons.

We next study the surface charging effect on the MAE of a system. The magnetic moments and MAE of a system with respect to charging are shown in Figure 3a,b, respectively. At zero charge, the magnetic moment is 3.012μ_B_ and MAE reaches its maximum value (~71.2 meV with an in-plane axis of magnetization). The magnetic moment and MAE remarkably reduce by adding electrons. Bader analysis shows that the electron charge of Re is in the range of 5.74~6.02e, which is 5.64e in the neutral form. This indicates only a small part of the injected electron charge transfers to the Re atom, and more electrons transfer to C atoms than neutral atoms, which may be visualized from the charge density difference under −0.2e per unit cell (see Figure 2c,d). The addition of electrons to the Re atom led to more pairing for spin-down electrons of d_z_^2^ orbitals, and then the magnetic moments of Re decrease. Removing the electrons from a system makes the magnetic moment of Re increase but the MAE reduce, accompanying the sign change. Bader analysis suggests that the electrons of Re are about 5.21e~5.55e, which decreases the spin-down electron of the d_yz_ pair and enhances the magnetic moment of Re. As mentioned above, the MAE of a system strongly depends on the Δl value. Figure 3c gives the Δl value with respect to charging, and one can see that the injecting charges led to a Δl decrease, except for −0.2e per unit cell. On the whole, small Δl may result in small MAE, which indicates that the Bruno relationship is still valid [45].

**Figure 4:**
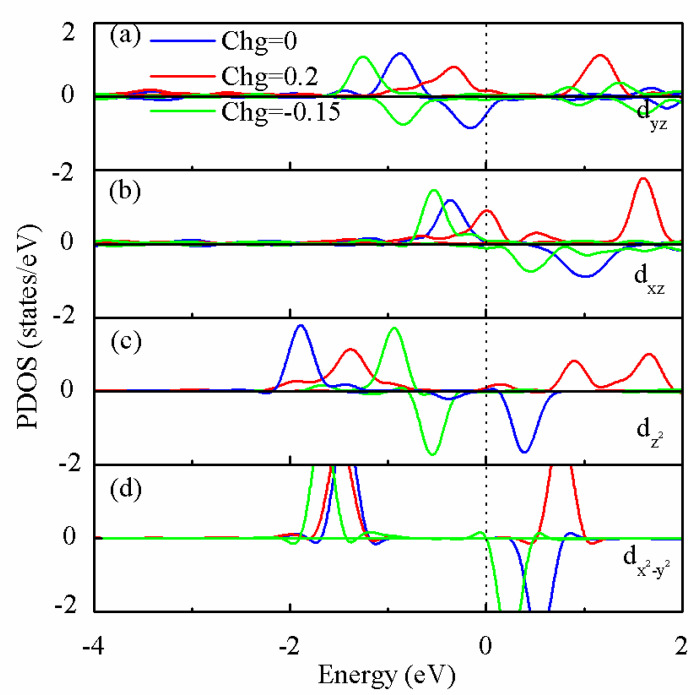
The projected density of states (PDOS) of typical Re-5d orbitals in Re@NDV under q = −0.15e, 0e and 0.2e injecting charge per unit cell, respectively. (**a**) d_yz_ orbital, (**b**) d_xz_ orbital, (**c**) d_z_^2^ orbital, (**d**) d_x^2^−y^2^_ orbital.

**Figure 5:**
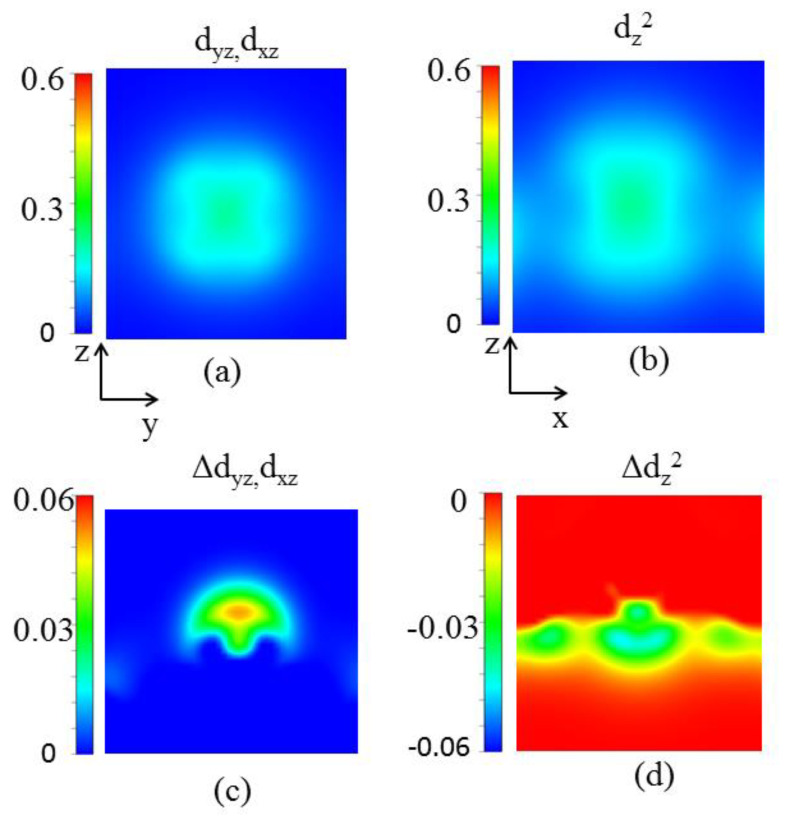
(Color online) (**a**,**b**) Surface charge densities of d_z_^2^ and (d_xz_,d_yz_) states, respectively, under neutral near the Fermi level. (**c**,**d**) Charge density differences induced by removing 0.2e per unit cell ($∆\rho =\rho \left(0.2e\right)-\rho \left(0e\right)$. The unit of contour values is e/Å^3^.

To illuminate the origin of manipulating MAE by injecting charge, Figure 4 plots the projected density of states (PDOS) of Re-5d orbitals with charging of −0.15e and 0.2e per unit cell, respectively. Based on the second-order perturbation formula [1,40], the MAE value can be expresses as

(2) $$\mathrm{MAE}=\xi^{2}\underset{\mu ,ο,\alpha ,\beta }{\sum }\left(2\delta_{\alpha \beta }-1\right)\left[\frac{{\left|\langle \mu ,\alpha \left|L_{x}\right|ο,\beta \rangle \right|}^{2}-{\left|\langle \mu ,\alpha \left|L_{y}\left(L_{z}\right)\right|ο,\beta \rangle \right|}^{2}}{\varepsilon_{\mu ,\alpha }-\varepsilon_{\mu ,\beta }}\right],$$

where *ξ* is the coefficient of SOC, and *μα* and *οᵦ* are the energy levels of the unoccupied spin *α* and occupied spin *β* state, respectively. From the denominator of Equation (2), it can be seen that the most dominant contribution to MAE comes from the orbitals around the Fermi level. As displayed in Figure 4, the main contribution to MAE is the coupling between spin-down occupied d_yz_ and spin-down unoccupied d_x_^2^_−y_^2^ (d_z_^2^) through the L_x_ operator in neutral. Adding 0.15 electrons per unit cell make spin-down d_yz_, d_x_^2^_−y_^2^ shift away the Fermi energy level and the unoccupied spin-down d_z_^2^ becomes the occupied state. MAE originates from the coupling between spin-down d_yz_ and d_x_^2^_−y_^2^ through the L_x_ operator. According to Equation (2), the states near the Fermi energy decrease, which is caused by adding electrons, leading to MAE reduction. When removing 0.2 electrons per unit cell from a system, spin-down d_z_^2^ and d_yz_ disappear, but new spin-up d_yz_ and d_xz_ appear near the Fermi energy level, which can be visualized by the charge density difference between 0e and 0.2e per unit cell. Figure 5a,b show the neutral surface charge densities of d_z_^2^ and (d_xz_,d_yz_) states near the Fermi level, respectively. The differences in the electron charge density between neutral and 0.2e of these orbitals are shown in Figure 5c,d, respectively. The positive and negative values of the charge density difference of d_xz_, d_yz_ and d_z_^2^ orbitals suggest that removing electrons induces a small d_z_^2^→(d_xz_,d_yz_) charge transfer. Because the SOC between the occupied spin-up d_yz_ and unoccupied d_xz_ states through the L_z_ operator plays an important role in negative contributions to MAE, variations in d_xz_, d_yz_ and d_z_^2^ occupation result in a change in easy magnetization direction and a reduction in the MAE of a system when removing electrons. Re@NDV is a good candidate for a magnetic storage device because it has large MAE and can be regulated by charge injection. Large MAE can ensure long-term stable storage of stored data, while small MAE can reduce energy consumption for writing information. In the future, it can be used as a storage medium for high-performance mobile devices.

## 4. Conclusions

We investigated the stability, magnetic properties and modification of Re@NDV using an applied external electric field and charging using DFT calculations. It was found that Re@NDV possesses high stability and large MAE. External electric fields do not influence the MAE much, while charge injection can bring dramatic changes to the magnitude of MAE and easy magnetization direction. The orbital-resolved PDOS demonstrates that there is critical variation in d_z_^2^ and d_yz_ of Re under an external electric field and charge injection, which is responsible for the change in MAE. The high-stability, large MAE and controllable MAE of Re@NDV means it has potential applications in high-performance magnetic storage and spintronics devices. With the continuous miniaturization of spintronic devices, it is crucial to improve magnetic storage density. In the future, we will explore two-dimensional magnetic materials with larger MAE to meet the needs of people’s practical applications and to regulate MAE through various methods, such as strain, adding an electric field, etc.

## Figures and Tables

**Table 1:** The distance (d) between the Re atom and graphene, binding energy (E_b_), the total magnetic moment (M_total_) the charge (Q) of Re atoms, and magnetic anisotropy energy (MAE) for Re@NDV and Re@DV. (Re@DV means double-divacancies graphene adsorbs Re atoms; Re@NDV means nitrogenized-divacancies graphene adsorbs Re atoms.)

Hybrid System	d (Å)	E_b_ (eV)	M_total_ (μ_B_)	Q_Re_	MAE (meV)
Re@NDV	0.30	6.94	3.02	1.36	71.20
Re@DV	0.78	8.10	1.05	1.06	2.67

## Data Availability

All data is contained within the manuscript.
